# Vulvar epithelioid leiomyoma with myxoid change: A case report and literature review

**DOI:** 10.1097/MD.0000000000017423

**Published:** 2019-10-18

**Authors:** Si-Hyong Jang, Hyun Deuk Cho, Ji-Hye Lee, Hyun Ju Lee, Soon Auck Hong, Hyein Ahn, Seong Taek Mun, Mee-Hye Oh

**Affiliations:** a Departments of Pathology, College of Medicine, Soonchunhyang University, Cheonan; b Departments of Pathology, College of Medicine, Chung-Ang University, Seoul; c Departments of Obstetrics and Gynecology, College of Medicine, Soonchunhyang University, Cheonan, Republic of Korea.

**Keywords:** epithelioid leiomyoma, smooth muscle tumor, vulvar neoplasms

## Abstract

**Rationale:**

Smooth muscle tumors of the vulva are infrequent neoplasms with diverse histologic features and unclear biologic behavior. Herein, we report a very rare case of vulvar epithelioid leiomyoma and review of previous reported cases of these tumors. In addition, we have discussed the representative diagnostic criteria of vulvar smooth muscle tumors and prognostic significance of epithelioid morphology.

**Patient concerns:**

We recently met a 45-year-old woman with complaint of painful vulvar mass.

**Interventions:**

Excisional biopsy was performed.

**Diagnoses:**

Pathologic examination revealed a vulvar epithelioid leiomyoma with multinodular growth pattern. Mitotic activity was rare and cellular atypia was not identified. Based on histology and immunohistochemical staining results, the case was diagnosed as vulvar epithelioid leiomyoma.

**Outcomes:**

After mass excision, the patient was discharged with no complication and there was no evidence recurrence for 6 months.

**Lessons:**

After reviewing previous papers and diagnostic criterion, we thought that vulvar smooth muscle tumors with predominant epithelioid morphology may be associated with unfavorable prognosis, Therefore, pathologists should examine the epithelioid component in vulvar smooth muscle tumors carefully.

## 1 Introduction

Smooth muscle tumors of the vulva are very uncommon entities and this rarity could lead to misdiagnosis of these tumors as other benign condition including Bartholin cysts or abscess.^[1,2]^ They are considered to originate from a variety of tissues in vulva including smooth muscle in erectile tissue, blood vessel walls, the round ligament, the dartos muscle or the erectorpili muscle.^[3,4]^ Histologically, smooth muscle tumors of the vulva typically display diverse morphologic features of spindled, epithelioid and myxohyaline and these components may appear either alone or mixed.^[4]^ Due to rare frequency and morphological complexity of these tumors, it is difficult to attain accurate diagnosis and predict the prognosis.

Although various histologic features can be observed, the diagnostic criterion is applied uniformly.^[3]^ In the present study, we report a rare case of vulvar leiomyoma with epithelioid and focal myxohyaline feature. Patient has provided informed consent for publication of this case. Also, we provide a review of the literatures of smooth muscle tumors of the vulva along the existing diagnostic criteria.

## 2 Case report

A 45-year-old woman visited the out-patient department for evaluation of a right vulvar mass with pain and tenderness. She incidentally recognized the tumor a few days back and the pain tended to aggravate during the period of menstruation. There were no other symptoms like fever, erythema and discharge. The past personal medical history was unremarkable. Physical examination revealed a mass on her right labia minor, measuring 1.7 × 1.4 cm in size. The tumor was superficially seated and movable. Ultrasound revealed a hypoechoic lesion that suggests Bartholin gland cyst or abscess. The uterus and both the adnexae were not significant. A mass excision was performed for definitive diagnosis and treatment. The vulvar mass was located in deep dermis and it grossly exhibited a tortuous appearance with soft rubbery consistency.

**Figure 1:**
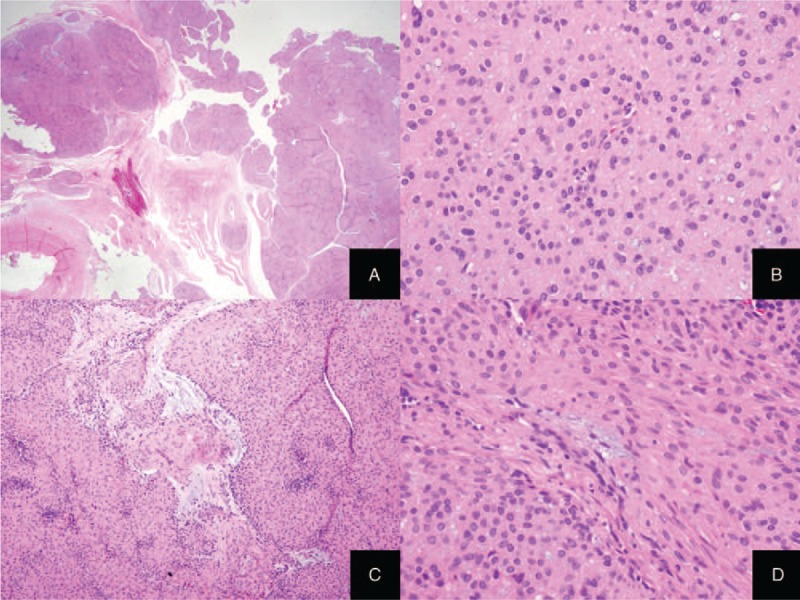
Microscopic features of vulvar mass. (A) The tumor showed multinodular growth pattern and well circumscription. (B) Tumor cells had epithelioid features with abundant eosinophilic cytoplasm and a round, centrally located nuclei lacking cellular pleomorphism or mitotic activity. (C) Stromal myxoid change was noted in some areas. (D) In some foci, the tumor consisted of typical bland-looking smooth muscle cells.

**Figure 2:**
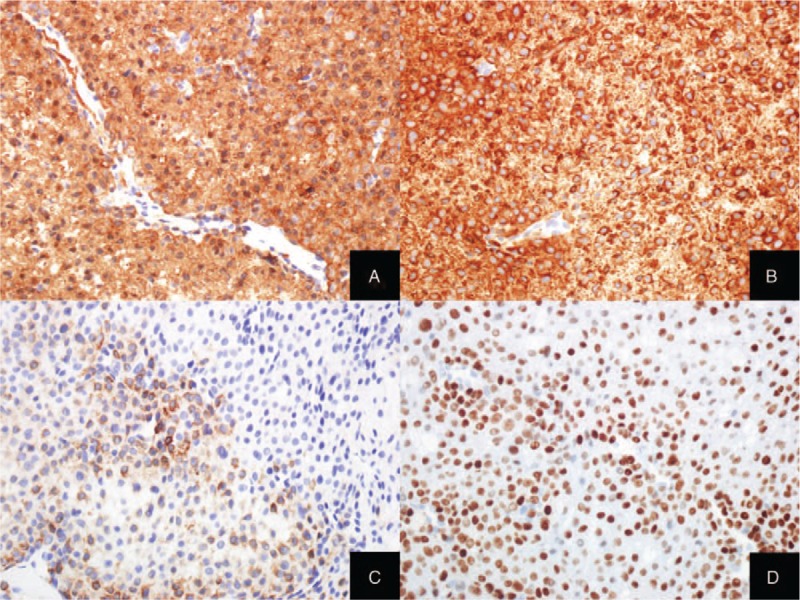
Immunohistochemical staining results in vulvar mass. Immunohistochemistry for SMA (A) and Smm-hc (B) was positive and immunohistochemistry for desmin (C) was focally positive. ER (D) was diffusely and strongly expressed in tumor cells.

Microscopic examination revealed a well-demarcated convoluted or multinodular tumor with solid growth pattern (Fig. 1A). The tumor comprised of mainly uniform epithelioid round or polygonal cells with abundant eosinophilic cytoplasm and centrally located round nuclei (Fig. 1B). Extracellular myxoid materials were multifocally identified in stroma and intracellular vacuolar myxoid change was noted (Fig. 1C). The focal areas were made up of spindle cells with blunt-ended elongated nuclei in part. (Fig. 1D). In some areas, the tumor originated from the vessel wall. Mitotic activity, cellular atypia or necrosis pleomorphism were absent. Immunohistochemistry revealed smooth muscle actin (SMA), smooth muscle myosin heavy chain (Smm-hc) and estrogen receptor (ER) as diffuse strong positive and focal positivity for desmin (Fig. 2A–D). HMB45 was not expressed in the tumor cells. Based on histologic features and immunohistochemistry, the tumor was diagnosed as vulvar epithelioid leiomyoma. The patient was discharged without any other complications and there was no evidence of clinical or radiologic recurrence for 10 months on a periodic follow-up.

## 3 Discussion

Vulvar leiomyomas are infrequent tumors accounting for 4.2% of cutaneous leiomyomas and 0.07% of all vulvar tumors.^[3]^ Among them, vulvar leiomyomas comprising of epithelioid cells as a dominant histologic component are of very rare occurrence.^[5]^ Vulvar epithelioid leiomyomas were first introduced in 1979 by Tavassoli and Norris.^[1]^ Earlier reports revealed that vulvar smooth muscle tumors with epithelioid feature showed a worse prognosis than the usual type of leiomyoma.^[1,6]^ Subsequently, additional cases were reported and to the best of our knowledge, 15 cases have been added in the English literature until date. The clinicopathologic characteristics of the previous cases and the present case are described in Table 1.

**Table 1:**
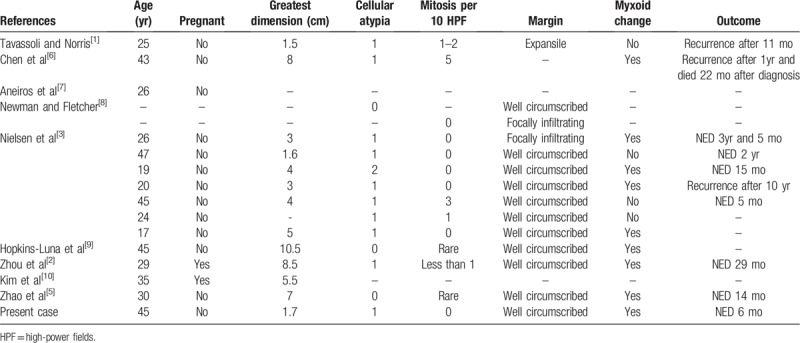
Clinicopathologic features of the epithelioid leiomyomas of the vulva.

The age when vulvar epithelioid leiomyomas tend to occur range from 19 to 47 years with a mean age of 31.7 years. In 2 patients, the tumor was detected during pregnancy. The size of the tumor varied from 1.5 cm to 10.5 cm in the largest dimension (mean tumor size: 4.87 cm).^[1–3,5–10]^ Regardless of the unique morphology, most of the cases exhibited no or minimal cellular pleomorphism. Moderate degree of cellular atypia was identified in only 1 report.^[3]^ In most of the cases, mitotic count was very low and five mitoses per 10 HPFs were observed in 1 case.^[6]^ Tumor margin was relatively well circumscribed or focally infiltrative in all the reported cases and myxoid stroma could be easily found in these tumors (9 out of 13 cases). Despite the benign histologic findings, frequent occurrence of local recurrence was noted (3 out of 9 cases). In the present case, the typical histologic features of vulvar epithelioid leiomyoma including absence of atypia, absence of mitosis and well circumscription were observed. Multifocal myxoid areas were also noted.

The diagnosis of smooth muscle tumors of the vulva is associated with a problem of establishing the diagnostic criterion which could differentiate between benign and malignant tumors. Tavassoli et al proposed three histologic factors to predict the prognosis of smooth muscle tumors of vulva. A large tumor size (>5 cm), an infiltrative margin and a high mitotic count (≥ 5 mitosis/10HPFs) were suggested as the predictive factors for recurrence and diagnostic features for sarcoma.^[1]^ Subsequently, Nielsen et al proposed an expanded and specific criterion based on the review of their 25 cases and the previously reported literatures. The researches gave attention to four characteristics including a large tumor size (≥5 cm), an infiltrative margin, a high mitotic activity (≥5 mitosis/10HPFs) and moderate to severe nuclear atypia. Nielsen et al categorized the tumors with 3 or 4 features as leiomyosarcoma, the tumors with 2 as atypical leiomyoma and the tumors with none or one as leiomyoma.^[3]^ Apparently, 3 out of 4 recurrent or metastatic smooth muscle tumors of the vulva reported in their article were classified as leiomyosarcoma, suggesting the appropriateness of the above criterion.

However, it remains whether the same criterion could be applied to smooth muscle tumors of the vulva with predominant epithelioid morphology. In previous reports on vulvar epithelioid leiomyomas, recurrence was observed in 3 out of 9 patients. One of the 3 recurrent cases fulfilled the requirements of being classified as atypical leiomyoma and remaining two cases met the criteria of leiomyoma category. Recurrence of vulvar epithelioid leiomyomas occurs in a range of 11 months to 10 years after diagnosis.^[1,3,6]^ Although there was no evidence of relapse for 10 months in the present case, a longer follow-up evaluation is needed. We thought that epithelioid histology of vulvar smooth muscle tumors may be associated with poor prognosis or frequent recurrence, but additional cases should be collected and studied.

In conclusion, we report a very uncommon case of vulvar smooth muscle tumor with a predominant epithelioid component along with a review of previous literatures. We have summarized the pathologic features and reviewed a criterion of smooth muscle tumors of the vulva. It is necessary to be aware of epithelioid morphology of vulvar smooth muscle tumors for presenting an appropriate diagnostic criterion to predict the prognosis.

## Author contributions

**Conceptualization:** Si-Hyong Jang, Mee-Hye Oh.

**Data curation:** Soon Auck Hong, Seong Taek Mun.

**Methodology:** Hyun Deuk Cho.

**Validation:** Hyun Ju Lee.

**Visualization:** Ji-Hye Lee.

**Writing – original draft:** Si-Hyong Jang, Hyein Ahn.

## References

[1] Tavassoli FA Norris HJ Smooth muscle tumors of the vulva. Obstet Gynecol 1979;53:213 7. 418977

[2] Zhou J Ha BK Schubeck D Myxoid epithelioid leiomyoma of the vulva: a case report. Gynecol Oncol 2006;103:342 5. 16765425 10.1016/j.ygyno.2006.04.001

[3] Nielsen GP Rosenberg AE Koerner FC Smooth-muscle tumors of the vulva. A clinicopathological study of 25 cases and review of the literature. Am J Surg Pathol 1996;20:779 93. 8669526 10.1097/00000478-199607000-00001

[4] Nucci MR Fletcher CD Vulvovaginal soft tissue tumours: update and review. Histopathology 2000;36:97 108. 10672053 10.1046/j.1365-2559.2000.00865.x

[5] Zhao T Liu X Lu Y Myxoid epithelial leiomyoma of the vulva: a case report and literature review. Case Rep Obstet Gynecol 2015;2015:894830. 26185695 10.1155/2015/894830 PMC4491551

[6] Chen KT Hafez GR Gilbert EF Myxoid variant of epithelioid smooth muscle tumor. Am J Clin Pathol 1980;74:350 3. 7416086 10.1093/ajcp/74.3.350

[7] Aneiros J Beltran E Garcia del Moral R Epithelioid leiomyoma of the vulva. Diagn Gynecol Obstet 1982;4:351 6. 7166124

[8] Newman PL Fletcher CD Smooth muscle tumours of the external genitalia: clinicopathological analysis of a series. Histopathology 1991;18:523 9. 1879812 10.1111/j.1365-2559.1991.tb01479.x

[9] Hopkins-Luna AM Chambers DC Goodman MD Epithelioid leiomyoma of the vulva. J Natl Med Assoc 1999;91:171 3. 10203920 PMC2608458

[10] Kim HR Yi BH Lee HK Vulval epithelioid leiomyoma in a pregnant woman. J Obstet Gynaecol 2013;33:210 1. 23445157 10.3109/01443615.2012.737051

